# Effect of Hydrothermal (Sr)-Hydroxyapatite Coatings on the Corrosion Resistance and Mg^2+^ Ion Release to Enhance Osteoblastic Cell Responses of AZ91D Alloy

**DOI:** 10.3390/ma13030591

**Published:** 2020-01-27

**Authors:** Chung-Wei Yang, Guan-Kai Wang

**Affiliations:** Department of Materials Science and Engineering, National Formosa University, No. 64, Wunhua Road, Huwei, Yunlin 63201, Taiwan; 10572105@gm.nfu.edu.tw

**Keywords:** biodegradable magnesium alloys, hydrothermal synthesis, nano-topography, corrosion resistance, dissolution behaviors, cell viability

## Abstract

The biomedical applications of Mg-based alloys are limited by their rapid corrosion rate in the body fluid. In this study, the hydrothermal synthesis is employed to produce protective bioactive hydroxyapatite coating (HAC) and strontium-substituted hydroxyapatite coating (Sr-HAC) to further enhance the corrosion resistance and in vitro biocompatibility of biodegradable AZ91D Mg alloy in physiological environments. For comparison, the brucite Mg(OH)_2_ prepared by the alkaline pre-treatment is designated as a control group. Experimental evidences of XRD and XPS analysis confirm that Sr^2+^ ions can be incorporated into HA crystal structure. It is noted that the hydrothermally synthesized Sr-HAC conversion coating composed of a specific surface topography with the nanoscaled flake-like fine crystallites is constructed on the AZ91D Mg alloy. The hydrophilicity of Mg substrate is effectively enhanced with the decrease in static contact angles after performing alkaline and hydrothermal treatments. Potentiodynamic polarization measurements reveal that the nanostructured Sr-HAC-coated specimens exhibit superior corrosion resistance than HAC and alkaline pre-treated Mg(OH)_2_. Moreover, immersion tests demonstrate that Sr-HAC provides favorable long-term stability for the Mg alloy with decreasing concentration of released Mg^2+^ ions in the SBF and the reduced corrosion rate during the immersion length of 30 days. The cells cultured on Sr-HAC specimens exhibit higher viability than those on the alkaline-pre-treated Mg(OH)_2_ and HAC specimens. The Sr-substituted HA coating with a nanostructured surface topography can help to stimulate the cell viability of osteoblastic cells.

## 1. Introduction

Magnesium (Mg) and its alloys are the candidates of the state-of-the-art new generation biodegradable metallic materials for the applications of cardiovascular devices, hard tissue replacements, regeneration therapies and bone graft scaffolds of tissue engineering [1,2,3,4,5,6]. In recent decades, Mg-based alloys have been widely studied and developed as orthopedic surgical implants due to their adequate mechanical properties, such as low density, high specific strength and low elastic modulus [7,8,9], which can effectively reduce the bone absorption and the stress shielding effect between Mg implants and the surrounding bone [10]. Moreover, Mg^2+^ ion is also a basic and beneficial cation for the human body to induce bone cell adhesion and the growth of new bone tissues with a firm bone-implant fixation for the implantation of Mg alloys [11,12].

However, the major drawback of Mg alloys is their chemically active properties in nature. An excessively high concentration of Mg^2+^ ions, which resulted from rapid corrosion behavior of Mg implants, induces the cytotoxic effect and inhibits the formation of extracellular matrix [13]. In addition, the mismatch between the degradation rate of Mg implants and the growth of new bone tissues may result in a prolonged healing process after implantation. The challenge of Mg-based implants is to have its degradability in a controlled manner. Since the appropriate concentration of Mg^2+^ ions play an essential role in bone remodeling [14], a controllable and protective coating is particularly needed for regulating the degradation rate of Mg alloys [15,16]. Therefore, some surface modification methods, such as chemical conversion coatings, chemical modification with organic compounds, electrochemical deposition, micro-arc oxidation and hydrothermal crystallization methods, are adopted to deposit a surface coating for improving the corrosion resistance and reducing the degradation rate of Mg alloys [17,18,19,20,21].

The main concept for preparing a protective surface coating on biodegradable Mg implants is to reduce the release of Mg^2+^ ions with proper corrosion resistance and to have biocompatibility with the surrounding hard tissues. Since calcium and phosphorus are the major elements in human bones, calcium phosphates (CaPs) are therefore widely selected as the surface protecting coatings of Mg-based alloys [21,22,23] due to their non-toxicity and excellent biocompatibility to the human body [24,25]. Synthetic hydroxyapatite (HA) is a bioceramic, which is chemically and crystallographically similar to the mineral constituents of hard tissues. HA is also the most famous bioactive and osteoconductive bioceramic among these CaP-series compounds. HA is commonly used as an implant coating to promote osseointegration, bone bonding ability and new bone growth on biomedical implants. Unlike synthetic HA, however, natural bone apatite is generally a non-stoichiometric crystal structure with the substitution of some trace cations or anions, such as Mg^2+^, Sr^2+^, Zn^2+^, CO_3_^2–^ and F^−^ ions, in human hard tissues [26]. It is demonstrated that the substitution of Mg^2+^, Zn^2+^ and F^−^ ions in HA can cause changes in the microstructure, further improve the degree of crystallinity, long-term chemical stability, promote cellular proliferation and reduce the solubility as well as the dissolution rate of ion-doped HA in physiological conditions with respect to the stoichiometric pure HA [27,28,29,30]. In addition, strontium (Sr) is a bone-seeking element, and it is recognized as one of the important trace elements found within human bones. Several studies have shown that the presence of Sr element has positive effects to enhance bone formation and regeneration, inhibit bone resorption and prevent osteoporosis [31,32,33]. Therefore, the deposition of Sr-containing bioactive HA protective coatings on Mg-based alloys would be beneficial for the enhancement of both biocompatibility and long-term stability of Mg implants with a suitable degradation rate.

In the present paper, alkaline pre-treated AZ91D Mg substrate, hydrothermally synthesized bioactive HA and Sr-containing HA coated AZ91D specimens were prepared to improve the chemical stability and biocompatibility and enhance in vitro corrosion resistance of biodegradable AZ91D Mg alloy based on the aforementioned concepts of depositing protective coatings on Mg implants for biomedical applications. The aim of the present study is to compare and determine the optimal characteristics and biological responses of surface coatings.

## 2. Materials and Methods

### 2.1. Preparation of the Hydrothermal Coatings

Commercial die-cast Mg alloy AZ91D sheets with chemical composition (in wt. %) of 8.93% Al, 0.69% Zn, 0.15% Mn, 0.0135% Si, <0.05% Fe, <0.03% Cu and balance Mg were used as substrates. AZ91D sheets were cut into rectangular specimens with dimensions of 30 × 10 × 3 mm for the hydrothermal coating process. The samples were ground with 1000-grit SiC abrasive papers, grit-blasted by Al_2_O_3_ particles (355–425 μm) to roughen the surfaces and then ultrasonically washed in ethanol and deionized water for 10 min to remove impurity particles. Alkaline treatment was recognized as an effective surface treatment to improve the electrochemical corrosion of Mg-based alloys [34]. Subsequently, the cleaned AZ91D were alkaline pre-treated in a 10 M NaOH solution at 70 °C to stabilize the oxides into a conversion surface layer; they will be designated as “AZ91D-AP”.

Analytical grade of CaHPO_4_·2H_2_O (DCPD, 172.09, AR, 98 wt. %, SHOWA, Osaka, Japan), Ca(OH)_2_ (74.09, AR, 96 wt. %, Berlin, Germany) and Sr(NO)_3_ (211.63, AR, 99 wt. %, ACROS, Geel, Belgium) were used as reagents for the hydrothermal synthesis of HA and Sr-substituted HA composite coatings. In order to confirm whether Sr element was substituted into HA structure, pure HA coating was prepared as a control for comparison. An aqueous solution containing powder mixtures of DCPD and Ca(OH)_2_ with deionized water was prepared with a controlled Ca/P molar ratio of 1.67 for hydrothermally synthesizing stoichiometric HA coatings; they will be designated as “HAC”. Subsequently, a 0.5 M Sr(NO)_3_ solution was added drop-wise into another prepared aqueous solution of DCPD and Ca(OH)_2_ mixtures with stirring for 15 min. The mixed solution with a (Ca,Sr)/P molar ratio of 1.67 was used as the reactant for hydrothermally synthesizing Sr-substituted HA coatings; these coated specimens will be denoted by “Sr-HAC” in the following. The hydrothermal treatment for synthesizing both HAC and Sr-HAC on AZ91D was performed at 175 °C, held for 2 h in an autoclave.

### 2.2. Specimen Characterization and Analysis

Surface morphologies, microstructural features, roughness and phase compositions were determined using a scanning electron microscopy (SEM) and a profilometer and X-ray diffraction (XRD), respectively. An X-ray diffractometer (XRD, Bruker, D8A25, Bruker Corp., Karlsruhe, Germany) was employed for the identification of phase compositions. The XRD analysis was conducted using Cu Kα radiation, operated at 40 kV and 40 mA with an incident angle of 5° and a scan speed of 3° (2θ) min^−1^ (step size, 0.02°). The surface microstructures of grit-blasted AZ91D, AZ91D-AP, HAC and Sr-HAC specimens were analyzed by scanning electron microscopy (SEM, JEOL/JSM-6360, JEOL Ltd., Tokyo, Japan). SEM equipped with an energy-dispersive X-ray spectroscopy (EDS) and electron probe micro-analyzer (EPMA, JEOL JXA-8530F, JEOL Ltd., Tokyo, Japan) was used to identify the elemental compositions and distributions. The surface roughness of the substrate and coatings was evaluated using a profilometer (Surfcorder SE-40G, Kosaka Laboratory, Tokyo, Japan). The measurement of static water contact angle was applied for wettability studies of the substrates with various surface coatings. Samples were cleaned with absolute alcohol, dried in N_2_ gas, and the contact angles were measured with a distilled water drop volume of 10 μL. The measurements were conducted in the air at room temperature.

The surface chemical compositions of alkaline pre-treated AZ91D-AP, hydrothermal HAC and Sr-HAC coatings were examined by X-ray photoelectron spectroscopy (XPS, PHI 5000 Versa Probe, ULVAC-PHI, Inc., Chigasaki, Kanagawa, Japan) using monochromatic Al Kα radiation (*hv* = 1486.7 eV). The base pressure of the XPS operating system was 1 × 10^−10^ Torr. As required, the measured binding energy (BE) was calibrated with reference to the adventitious C 1*s* peak at 284.6 eV. The chemical states and the substitution of Sr content were analyzed by XPS. The Gaussian peak-fitting routine was used in the analysis of high-resolution spectra for separating species in different chemical states.

### 2.3. Potentiodynamic Polarization and Static Immersion Tests in SBF

The in vitro electrochemical anti-corrosion behaviors of the AZ91D Mg alloy, alkaline pre-treated AZ91D-AP, hydrothermally synthesized HAC and Sr-HAC coated specimens were evaluated by electrochemical potentiodynamic polarization tests in a conventional three-electrode electrochemical cell using a computer-controlled potentiostat (SP-150, Bio-Logic Science Instruments, rue de Vaucanson, Seyssinet-Pariset, France) according to the ASTM G102-89. A platinum sheet and a saturated calomel electrode (SCE, Hg/Hg_2_Cl_2_) were used as the counter electrode and the reference electrode, respectively. Rectangular samples with an exposed area of 100 mm^2^ were applied as the working electrode. Measurements were performed at 37 °C in the standard simulated body fluid (SBF) at a constant scan rate of 0.5 mV/s. The SBF solution (pH = 7.4) was prepared according to ISO 23317 by the procedure of the Kokubo and Takadama’s recipe [35], in which the ion concentrations were similar to the human blood plasma. Corrosion current densities (*I*_corr._, μA/cm^2^) and corrosion potentials (*E*_corr._, V_SCE_) were extracted from potentiodynamic polarization curves by the Tafel slope extrapolation method. The instantaneous corrosion rate (CR, *P_i_*) evaluated by the corrosion current densities was determined from the equation *P_i_* = 22.85 × *I*_corr._ in mm/year. The corroded surface morphologies of AZ91D substrate and various surface treated specimens after potentiodynamic polarization tests were examined using SEM.

In order to examine long-term in vitro degradation behaviors of uncoated AZ91D, alkaline pre-treated AZ91D-AP, hydrothermally synthesized HAC and Sr-HAC specimens, static immersion tests were performed in SBF solution at 37 °C according to the ASTM G31-72. During the immersion period, a pH meter was used to record the pH value of SBF. First, the specimens were weighed before immersion, *W*_b_ (mg). After the immersion test, the specimens were taken out the SBF at each pre-defined time point. The concentrations of released Mg^2+^ and Al^3+^ ions from the AZ91D substrate into SBF solution were measured and analyzed by inductively coupled plasma-mass spectrometry (ICP-MS, ELEMENT XR, Thermo Scientific Inc., Waltham, MA, United States). Each specimen was washed with distilled water, dried for 24 h and weighed to determine the specimen weight, *W*_a_ (mg). The weight loss (mg cm^−2^ d^−1^) of specimens was then evaluated from the Equation (1):

(1) $$W_{L}=\frac{W_{b}-W_{a}}{A\cdot t}$$

where *A* was the surface area (cm^2^) of the specimen, and *t* was the immersion duration (d). The measurement of weight loss data for each immersion time was an average of five specimens (*n* = 5). The corresponding corrosion rate (*P_W_*, mm/year) evaluated by the immersion test was then determined from *P_W_* = 2.1 × *W_L_* [36].

### 2.4. In Vitro Osteoblast Cell Culture and Osteoblast Cell Viability Assay

The in vitro cell viability of cultured osteoblastic MG63 cells on the uncoated AZ91D substrate, alkaline pre-treated AZ91D, bioactive HAC and Sr-HAC coated specimens were assessed in this study. Human osteoblast-like MG63 cells purchased from the American Type Culture Collection (ATCC, Rockville, MD, USA) were performed to investigate the effects of above-mentioned surface protective coatings and ion release on the viability of osteoblastic cells. The culture medium for the MG63 cells was Dulbecco’s modified Eagle’s medium (DMEM, Gibco, Waltham, Massachusetts, USA) supplemented with 10% fetal bovine serum (FBS, Sigma-Aldrich, Germany) and 1% penicillin-streptomycin (Life Technology, Munich, Germany). The cells were incubated at 37 °C in an atmosphere of 95% humidified air and 5% CO_2_. The culture mediums were changed every 2 days, and the cells were washed with a phosphate-buffered saline (PBS) with 0.05% trypsin solution. All the specimens were sterilized at 180 °C for 2 h for the dry-heat sterilization. After sterilization, the specimens were placed in a 24-well culture plate at a seeding density of 1 × 10^4^ cells mL^−1^ for the test.

An indirect cytotoxicity assay was performed with the Mg^2+^ conditioned culture media collected from the incubation of the specimens in complete culturing medium without cells. Mg^2+^ conditioned media were collected for 1, 3 and 7 days. Pure cell culture medium without Mg^2+^ extract was used as the control. MG63 cell viability in the Mg^2+^ conditioned medium and on the specimens was determined by the MTT assay. Cells at a density of 1 × 10^4^ cells mL^−1^ were seeded on the culture plates. The culture medium was changed with the Mg^2+^ conditioned medium after cells attached. Then the indirect cytotoxicity assay was performed for cells cultured in days 1, 3 and 7 in the Mg^2+^ conditioned culture medium for 24 h. In addition, the cells at a density of 1 × 10^4^ cells mL^−1^ were seeded onto the sterilized specimens. The culture medium was changed every 2 days during the culture duration. After 1, 3 and 7 days of incubation, the culture medium was removed from each well, and the specimens were transferred to new 24-well culture plates. Subsequently, 200 μL of 10% 3-[4,5-dimethylthiazol-2-yl]-2,5-diphenyltetrazolium bromide (MTT, Gibco, Waltham, Massachusetts, USA) working solution were added to each well. MTT working solution was prepared by dissolving MTT in PBS at 5 mg/mL. The plates were then incubated for 4 h at 37 °C and 5% CO_2_. At the end of incubation, the medium in each well was replaced by 400 μL of dimethyl sulfoxide (DMSO, Sigma, Taufkirchen, Germany) to dissolve the formazan crystals. After shaking for 30 min, absorbance of the solution in each well was evaluated at a fixed wavelength of 570 nm using an enzyme-linked immunosorbent assay (ELISA) microplate reader (Sunrise, Tecan Trading AG, Männedorf, Switzerland), and the viability was calculated. The viability of cells was quantified by measuring the optical density (OD) value. The experiments were repeated independently 3 times. The cell viability data were expressed as the mean ± standard deviation (SD). Statistically significant differences were analyzed by one-way analysis of variance (ANOVA) at an average of at least triplicates, and the significance level was defined as *p* < 0.05.

## 3. Results and Discussion

### 3.1. Phase Composition and Surface Characteristic Analysis

**Figure 1:**
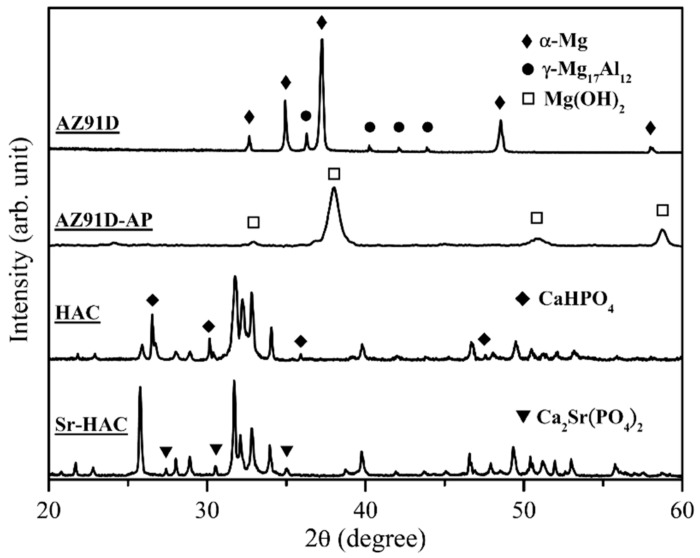
X-ray diffraction patterns of the AZ91D Mg alloy, alkaline pre-treated AZ91D-AP, hydrothermally synthesized hydroxyapatite coating (HAC) and strontium-substituted hydroxyapatite coating (Sr-HAC) surface coatings.

Figure 1 shows the X-ray diffraction (XRD) patterns of the grit-blasted AZ91D substrates, alkaline pre-treated AZ91D-AP specimens, hydrothermally synthesized HAC and Sr-HAC coatings.

The XRD pattern of AZ91D alloy displays typical diffraction peaks of the α-Mg phase (diffraction peaks at 2θ = 32.6, 34.9, 37.2, 48.5 and 58.0°, JCPDS 35-0821). In addition, the γ-Mg_17_Al_12_ phase (main peak detected at 2θ = 36.1°, minor peaks detected at 2θ = 40.1, 41.9 and 43.7°, JCPDS 73-1148), which is a common intermetallic compound (IMC) for the Mg-Al series alloys, is identified in the XRD pattern of AZ91D alloy. The XRD pattern of alkaline pre-treated AZ91D-AP specimens shows typical diffraction peaks of the brucite phase Mg(OH)_2_ (main peaks detected at 2θ = 38.0, 50.9 and 58.6°, a minor peak at 2θ = 32.8°, JCPDS 07-0239). It represents that a brucite Mg(OH)_2_ conversion surface layer is deposited on the Mg alloy after the alkaline pre-treatment. Phase composition of hydrothermally synthesized HAC specimens is predominantly composed of the hydroxyapatite phase (main peaks of (211), (112) and (300) crystal planes detected at 2θ = 31.77, 32.20 and 32.90°, respectively, JCPDS 09-0432) and the monetite phase CaHPO_4_ (dicalcium phosphate anhydrous, DCPA, main peaks detected at 2θ = 26.6° and 30.2°, minor peaks at 2θ = 35.9° and 47.5°, JCPDS 89-5969). The residual monetite CaHPO_4_ (DCPA) can be recognized as an intermediate phase during the hydrothermal synthesizing process of HA [37,38]. Compared with the XRD pattern of HAC specimens, hydrothermally synthesized Sr-HAC illustrates a well-crystallized Sr-substituted hydroxyapatite phase (Ca_8.98_Sr_1.02_(PO_4_)_6_(OH)_2_, main peaks of (211), (112) and (300) crystal planes detected at 2θ = 31.71, 32.09 and 32.86°, respectively, JCPDS 89-5631). It is noted that the substitution of cations or anions into HA crystal structure can be determined by the shift of (003) diffraction peak [27,39]. Since the ionic radius of Sr^2+^ ions (1.18 Å) is larger than that of Ca^2+^ ions (1.00 Å) [40], the substitution of Sr^2+^ ions for Ca^2+^ ions will cause a lattice expansion with the increase of lattice constants (*a* = 9.435, *c* = 6.908 for the Ca_8.98_Sr_1.02_(PO_4_)_6_(OH)_2_, *a* = 9.418, *c* = 6.884 for the Ca_10_(PO_4_)_6_(OH)_2_). Therefore, the expansion of apatite crystal lattice results in a slight shift of the characteristic (300) diffraction peak from 2θ = 32.90° for the HAC to smaller diffraction angle of 2θ = 32.86° for the Sr-HAC as a result of the XRD analysis. In addition, some minor diffraction peaks of the calcium strontium phosphate phase (β-Ca_2_Sr(PO_4_)_2_, diffraction peaks at 2θ = 27.4, 30.5 and 35.0°, JCPDS 52-0467) are also detected in the Sr-HAC XRD pattern, as indicated by the triangular marks in Figure 1. It can be recognized that the substitution of Sr^2+^ ions is beyond the solubility of Sr in the apatite crystal lattice [41,42], to further stabilizes and induces the formation of Sr-substituted tricalcium phosphate phase (β-Sr-TCP, β-Ca_2_Sr(PO_4_)_2_) during the synthesizing process.

**Figure 2:**
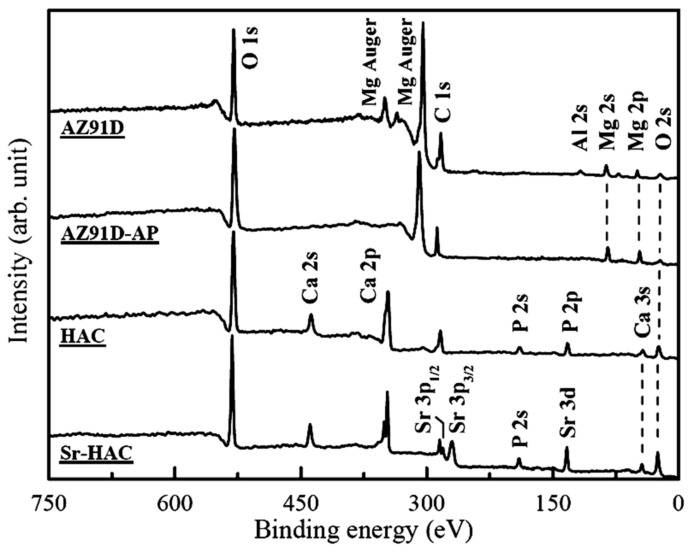
XPS survey spectra analysis for the AZ91D alloy, AZ91D-AP, HAC, Sr-HAC specimens.

The surface chemical states of AZ91D alloy with various surface treatments are determined by XPS analysis, and Figure 2 shows the survey XPS spectra of these specimens. It represents that the signals of C, O, Mg and Al elements are detected on the surface of AZ91D and AZ91D-AP specimens. The signals of Ca, Sr, P and O elements are detected on the HAC and Sr-HAC coatings.

**Figure 3:**
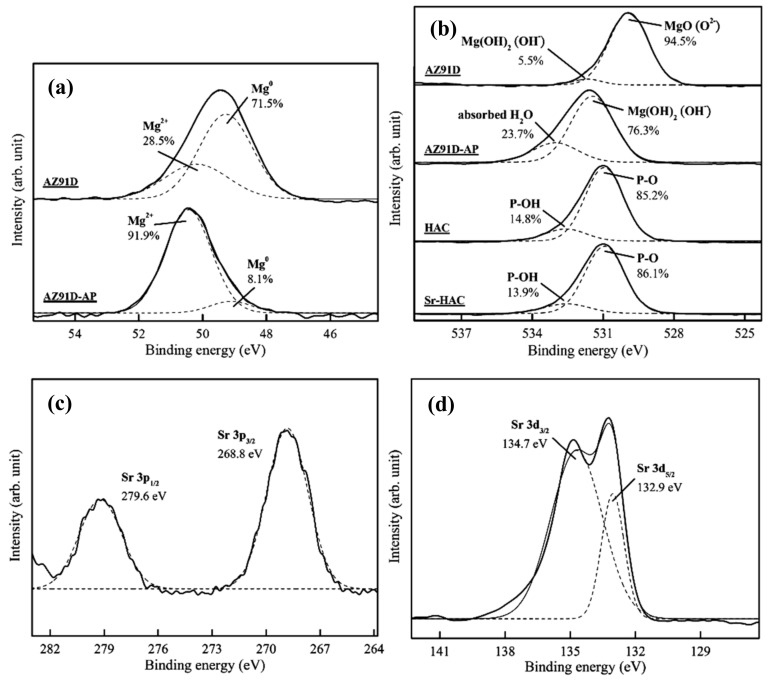
High-resolution XPS spectra of (**a**) Mg 2*p*, (**b**) O 1*s*, (**c**) Sr 3*p* and (**d**) Sr 3*d* bands for the Sr-HAC specimen with curve-fitting analysis results.

Figure 3 shows the high-resolution Mg 2*p*, O 1*s*, Sr 3*p* and Sr 3*d* XPS spectra. Figure 3a shows a representative high-resolution Mg 2*p* band and curve-fitting results of the AZ91D Mg alloy and AZ91-AP specimens. Considering the untreated AZ91D substrate, the Mg 2*p* band is deconvoluted into two peaks at binding energy (BE) about 49.3 eV and BE = 50.3 eV corresponding to metallic magnesium (Mg^0^, 71.5%) and Mg^2+^ ions (28.5%) in native magnesium oxide (MgO) or hydroxide (Mg(OH)_2_), respectively [43]. After performing the alkaline pre-treatment on AZ91D Mg alloy, the significant increasing intensity and integration area of the Mg^2+^ ions bonding peak from 28.5% to 91.9% can be attributed to the formation of brucite Mg(OH)_2_ surface layer (see Figure 3a cf. Figure 1) on AZ91D Mg alloy. Figure 3b shows representative high-resolution O 1*s* bands and curve-fitting results of the AZ91D, AZ91D-AP, HAC and Sr-HAC specimens. The corresponding O 1*s* band of the AZ91D substrate consists of two components at BE = 530.0 eV and BE = 531.5 eV, which correspond to the surface native magnesium oxide (MgO) and hydroxyl groups (OH¯) of the magnesium hydroxide (Mg(OH)_2_), respectively. As a result of Figure 3a,b, it can be recognized that MgO is the major product on the grit-blasted surface of Mg alloy. After the alkaline pre-treatment, the O 1*s* band of AZ91D-AP can be deconvoluted into two peaks at BE = 531.5 eV and BE = 533.0 eV corresponding to the OH¯ groups in hydroxide (Mg(OH)_2_) and surface absorbed H_2_O [44], respectively. The significantly increasing integration area of OH¯ groups in hydroxide (from 5.5% to 76.3%) results from the formation of Mg(OH)_2_ surface layer on AZ91D Mg alloy after the alkaline treatment. Considering the O 1*s* band of HAC and Sr-HAC specimens, the deconvoluted peaks located at about BE = 531.0 eV and BE = 532.5 eV represent the presence of P-O bond in phosphate groups (PO_4_^3–^) and P-OH bond of the apatite crystal structure, respectively [44]. As a result, similar peak intensity and integration area percentage of P-O and P-OH bonds represent that hydrothermally synthesized HAC and Sr-HAC coatings both exhibit well crystallized apatite crystal structure. Moreover, XPS analysis can be used to evaluate the substitution of Sr^2+^ ions on the chemical state of the Sr element in the coatings. The corresponding Sr 3*p* band of the Sr-HAC specimens presented in Figure 3c consists of two components at BE = 279.6 eV and BE = 268.8 eV, which correspond to the Sr 3*p*_1/2_ and the Sr 3*p*_3/2_ bands, respectively. The Sr 3*d* band is deconvoluted into two peaks at about BE = 134.7 eV and BE = 132.9 eV corresponding to Sr 3*d*_3/2_ and Sr 3*d*_5/2_ bands (see Figure 3d), respectively [45]. Based on the XPS analysis results, the detected Sr 3*p* and Sr 3*d* bands confirm that Sr^2+^ ions can be substituted into the HA crystal structure by the hydrothermal synthesizing process. The measured molar ratios of Sr/(Ca+Sr) and (Ca+Sr)/P are about 0.096 and 1.623, respectively. Therefore, the chemical composition of Sr-HAC can be expressed in the formula of Ca_9_Sr(PO_4_)_6_(OH)_2_, which is close to the identified phase composition through the XRD analysis.

### 3.2. Microstructural Features and Wettability of the Surface

**Figure 4:**
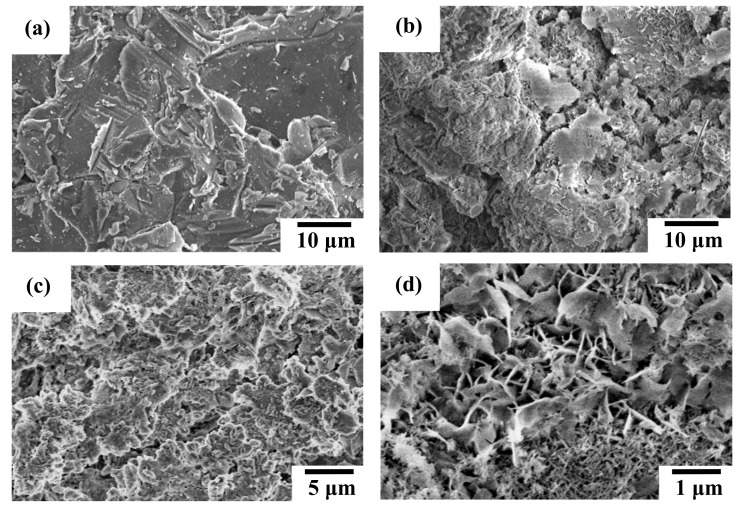
SEM surface morphologies of the (**a**) grit-blasted AZ91D substrate, (**b**) alkaline pre-treated AZ91D-AP, (**c**) hydrothermally synthesized HAC, and (**d**) Sr-HAC coatings.

Figure 4 shows the representative SEM micrographs of the AZ91D Mg alloy, AZ91D-AP, HAC and Sr-HAC specimens. Table 1 lists the measured average surface roughness (Ra, μm, *n* = 5) of these specimens. The grit-blasted AZ91D substrate displays a typical irregular surface with scratched grooves and craters, as illustrated in Figure 4a. The surface roughness of grit-blasted AZ91D is about 4.5 ± 0.2 μm. After performing various surface treatments, it can be seen that AZ91D-AP, HAC and Sr-HAC specimens display quite different microstructural features. Figure 4b shows the surface morphology of alkaline pre-treated AZ91D-AP specimens. It represents an irregular and much rougher surface topography (Ra = 11.5 ± 0.7 μm) of the agglomerated Mg(OH)_2_ conversion layer accompanied with obvious surface pores and cracks. The surface morphology of hydrothermally synthesized HAC coating is shown in Figure 4c. Compared with the AZ91D-AP specimens, we can see the HAC coating apparently displays less surface defects and lower surface roughness (Ra = 6.1 ± 0.5 μm). HA coating can be deposited more uniformly on the AZ91D substrate by the hydrothermal synthesizing process. It is noted that a specific surface morphology with nanoscaled flake-like fine crystallites is observed on the hydrothermally synthesized Sr-HAC coated AZ91D specimens, as illustrated in Figure 4d. Related studies have reported that a nanoscaled flake-like or needle-like crystal structure, highly crystalline, can be obtained by the hydrothermal process [46,47].

**Figure 5:**
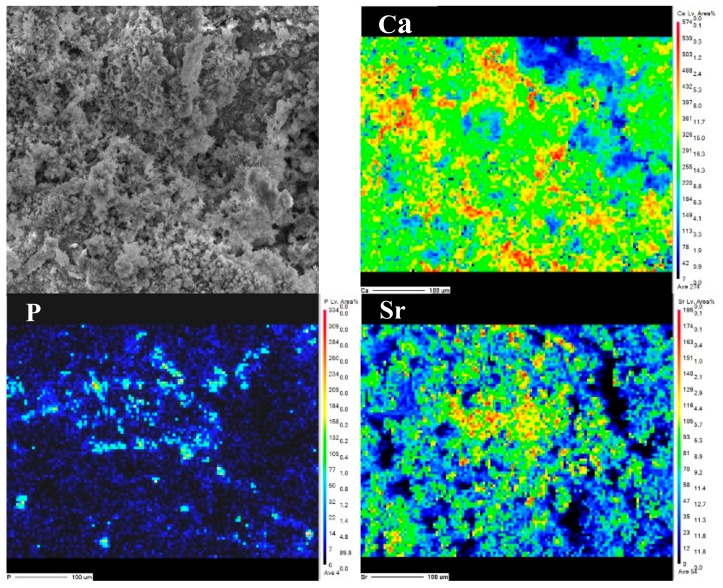
The EPMA analysis for Ca, P and Sr element distribution on the surface of Sr-HAC.

Figure 5 also represents the SEM image and EPMA analysis results for the element distribution of Ca, P and Sr on the surface of Sr-HAC. Referring to the XRD and XPS analysis results (see Figure 1 and Figure 3 cf. Figure 5), it is demonstrated that Sr^2+^ ions can be effectively substituted and uniformly distributed in the apatite lattice to form Sr-HAC coating. In addition, the Sr-HAC coating with a nanoscaled crystalline structure possesses significant much lower surface roughness (Ra = 2.7 ± 0.3 μm) than the other specimens. Several reports indicated that the nanoscaled crystalline surface is important for promoting bioactivity, in vivo osseointegration and cellular biocompatibility of implants [21,48] to be used for bone replacement and regeneration. Thus, it can be expected that the hydrothermally crystallized Sr-HAC with microstructures represented in Figure 4d will further assist in enhancing cell responses of the Sr-HAC coated Mg implants.

**Figure 6:**
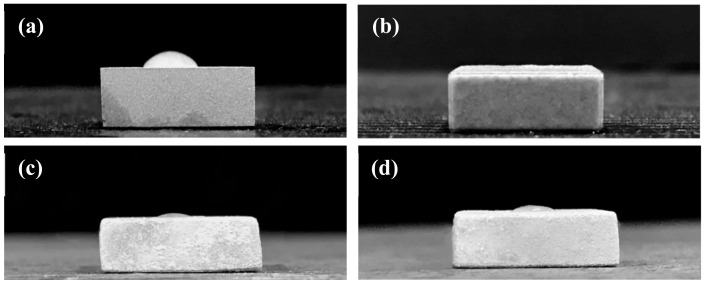
Hydrophilicity of the (**a**) grit-blasted AZ91D Mg alloy, (**b**) alkaline pre-treated AZ91D-AP, hydrothermally synthesized (**c**) HAC and (**d**) Sr-HAC specimens.

Moreover, the variation of surface wettability also directly affects the biocompatibility of implants in physiological environments. Figure 6 shows images of distilled water droplets on the surface of grit-blasted AZ91D substrate, alkaline pre-treated AZ91D-AP, hydrothermally synthesized HAC and Sr-HAC specimens by the static contact angle measurement. Table 1 also lists the static contact angles of various specimens. As a result, the static contact angle of water droplets on the grit-blasted AZ91D Mg alloy is about 55°, as illustrated in Figure 6a. After performing various surface treatments, we can see the hydrophilicity of the AZ91D Mg alloy is effectively enhanced with the decrease in static contact angles for AZ91D-AP, HAC and Sr-HAC specimens, as shown in Figure 6b–d. The hydrophilic surface will be beneficial to the cell adhesion, and the improvement of surface wettability can also promote high levels of early cells attachment after implantation [49]. The water contact angles of HAC and Sr-HAC coatings are about 10 and 12°, respectively. It is noted that the static contact angle of water droplets on the AZ91D-AP surface is almost reduced to about 0°, as shown in Figure 6b. Referring to the XRD and XPS analysis results represented in Figure 1 and Figure 3, brucite Mg(OH)_2_ and apatites (Ca_10_(PO_4_)_6_(OH)_2_ and Ca_9_Sr(PO_4_)_6_(OH)_2_) are the main phase compositions for AZ91D-AP, HAC and Sr-HAC specimens. Since the polar hydroxyl groups (OH^−^),supplied from the crystal structures of Mg(OH)_2_ and apatite phases, effectively induce an attractive interaction between these surface coatings and water molecules through the hydrogen bonds, it is recognized that the typical hydrophilic surfaces of AZ91D-AP, HAC and Sr-HAC coated AZ91D result from their polar surface chemistry. Meanwhile, the alkaline pre-treated AZ91D-AP specimen displays an irregular and much rougher surface topography accompanied with obvious pores and cracks (see Table 1 and Figure 4b). Therefore, the surface of alkaline pre-treated AZ91D-AP displays a super-hydrophilic state, which results from the strong polar surface chemistry of Mg(OH)_2_ layer and the capillary action of a rough surface morphology [50].

### 3.3. Electrochemical Corrosion Performances

**Figure 7:**
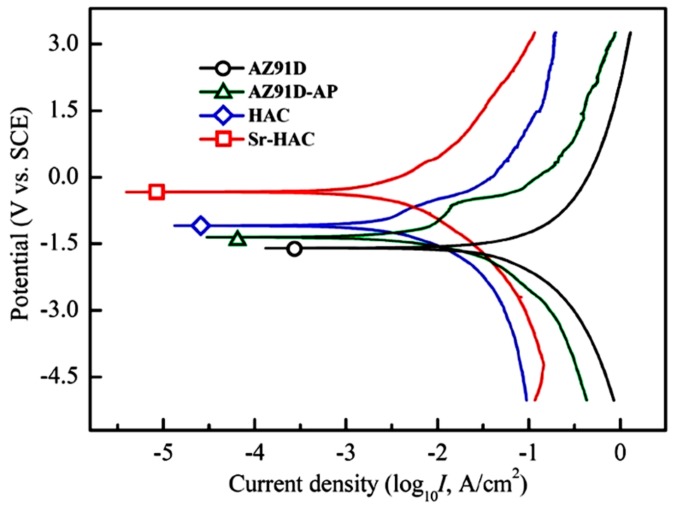
Potentiodynamic polarization curves of the AZ91D alloy, alkaline pre-treated AZ91D-AP, hydrothermally synthesized HAC and Sr-HAC specimens tested in Kokubo’s SBF solution at 37 °C.

Potentiodynamic polarization curves will provide information on the corrosion behaviors. Figure 7 shows the corrosion test results in terms of the potentiodynamic polarization curves of the AZ91D Mg alloy, AZ91D-AP, HAC and Sr-HAC specimens in the SBF solution at 37 °C. The values of corrosion potential (*E*_corr._, V_SCE_) and the estimated corrosion current density (*I*_corr._, μA/cm^2^) are obtained through the Tafel extrapolation method. The corrosion current density is calculated from the polarization resistance (*R*_p_, kΩ-cm^2^) by the Stern–Geary equation.

(2) $$I_{corr.}=\frac{\beta_{c}\beta_{a}}{2.3\cdot R_{p}(\beta_{c}-\beta_{a})}$$

Parameters *β*_a_ and *β*_c_ are the anodic Tafel slope and cathodic Tafel slope, respectively. The instantaneous corrosion rate (*P*_i_, mm/year) is determined from the corrosion current density using the following equation of *P*_i_ = 22.85 × *I*_corr_. Table 2 lists the corrosion parameters obtained from potentiodynamic polarization tests in the standard Kokubo’s SBF solution. As shown in Figure 7 and Table 2, the AZ91D substrate exhibits the most negative corrosion potential (*E*_corr._ = −1.58 V vs. SCE) of all the specimens. Generally, a shift of the corrosion potential toward the positive side, an increase of the polarization resistance and a decrease of the current density represent that corrosion reactions of the metallic substrate can be further reduced through performing various surface treatments. Therefore, we can see the corrosion potential of AZ91D-AP slightly shift to about −1.42 V, and the polarization resistance of the AZ91D substrate (*R*_p_ = 0.75 kΩ-cm^2^) is also obviously increased with the improvement of corrosion resistance by the alkaline pre-treatment (AZ91D-AP, *R*_p_ = 3.28 kΩ-cm^2^). The reduction of electrochemical corrosion reactions of the AZ91D substrate can also be demonstrated by the evident decrease of the corrosion current density from 96.8 μA/cm^2^ (uncoated AZ91D Mg alloy) to 20.2 μA/cm^2^ (AZ91D-AP specimens). It is worth noting that the corrosion potentials of the hydrothermally synthesized HAC and Sr-HAC specimens are both significantly increased to −1.05 V and −0.33 V, respectively. Meanwhile, the corrosion current density is also dramatically decreased from 96.8 μA/cm^2^ of the AZ91D substrate to 7.9 μA/cm^2^ of the HAC and 5.6 μA/cm^2^ of the Sr-HAC specimens (see Table 2). The Sr-HAC specimens display the highest corrosion potential and the lowest corrosion current density of all the conditions. In addition, the polarization resistances of HAC and Sr-HAC specimens are significantly improved to about 9.31 kΩ-cm^2^ and 12.86 kΩ-cm^2^, respectively. The values are obviously higher than that of the AZ91D (0.75 kΩ-cm^2^) and the AZ91D-AP specimens (3.28 kΩ-cm^2^). As a result of the potentiodynamic polarization tests, the presence of either AZ91D-AP, or the deposition of hydrothermally synthesized bioactive HA and Sr-HAC surface coatings, can lead to a substantial decrease in the instantaneous degradation rate of AZ91D Mg alloy within corrosive physiological environments. Since the improvement of the polarization resistance results from the protective effect of surface layers against corrosive solutions for the substrate, it can be concluded that hydrothermally synthesized HAC and Sr-HAC coatings provide much desirable protective effects to suppress corrosion reactions of AZ91D Mg alloy than alkaline pre-treated AZ91D-AP specimens.

**Figure 8:**
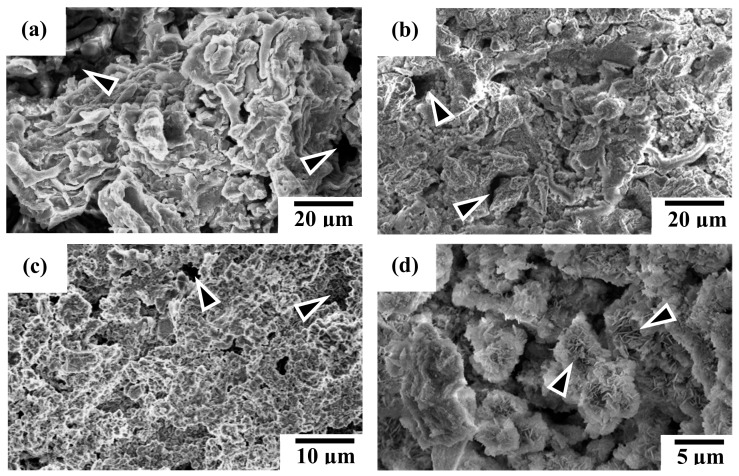
SEM corroded surface morphologies of the (**a**) AZ91D Mg alloy, (**b**) AZ91D-AP, (**c**) HAC, and (**d**) Sr-HAC coatings after potentiodynamic polarization tests.

Figure 8 shows the SEM micrographs of specimen surface after potentiodynamic polarization experiments. Figure 8a,b illustrates the corroded surface morphologies of AZ91D substrate and alkaline pre-treated AZ91D-AP specimens, respectively. The corroded surface morphology of AZ91D-AP specimens is similar to that of the uncoated AZ91D Mg alloy. In addition, obvious pitting corrosion regions can be usually observed on the corroded surface for both AZ91D and AZ91D-AP specimens, as those indicated by the triangular marks in Figure 8a,b. It is well-known that Mg(OH)_2_ is a common corrosion product of the Mg alloys, and Mg(OH)_2_ is slightly soluble in water. However, when the corrosive solution contains chloride ions (Cl^−^) with concentration exceeding 30 mmol/L [1], the AZ91D Mg substrate and its native Mg(OH)_2_ corroded surface layer will start to react with Cl^−^ ions to form highly soluble magnesium chloride (MgCl_2_). As a result, severe pitting corrosion of AZ91D occurs within the simulated body fluid of a high Cl^−^ ion concentration. Referring to the results of potentiodynamic polarization tests, the electrochemical performances and degradation rate of AZ91D-AP specimens in SBF solution appear to be slightly improved with the formation of a brucite Mg(OH)_2_ surface layer after performing the alkaline pre-treatment to the AZ91D Mg alloy (see Figure 1). However, the appearance of obvious pitting corrosion for AZ91D-AP (as indicated by the triangular marks in Figure 8b) shows that the alkaline pre-treated Mg(OH)_2_ with a rough topography and apparent surface defects (see Figure 4b) is insufficient to provide a desirable anti-corrosion performance to the AZ91D Mg alloy within the SBF solution. Thus, it is important to enhance the pitting corrosion resistance for Mg alloys as biomedical implanted materials.

Figure 8c shows the corroded surface morphology of hydrothermally synthesized HAC specimens for illustration. Comparing Figure 8c with Figure 4c, we can see less variation is observed on the corroded surface for the HAC specimens after electrochemical polarization tests. Moreover, the distribution of pitting corrosion regions on HAC is reduced and less than that on AZ91D-AP specimens, as those indicated by the triangular marks in Figure 8c. Therefore, the reduction of corrosion current density (*I*_corr._ = 7.9 μA/cm^2^) means that the corrosion reactions and the degradation/corrosion rate (*P*_i_ = 0.18 mm/year) of AZ91D alloy can be further suppressed by the deposition of HAC coatings. The increasing polarization resistance (*R*_p_ = 9.31 kΩ-cm^2^) also shows that the formation of HAC can provide better corrosion resistance than alkaline pre-treated Mg(OH)_2_ layer to impede the penetration of corrosive SBF solution to the Mg substrate. Figure 8d shows the corroded surface morphology of hydrothermally synthesized Sr-HAC specimens for illustration. It is worth noting that the corroded surface of Sr-HAC coating not only possesses structural integrity similar to that of HAC specimens but also the specific nanoscaled flake-like fine crystallites still remained on the surface (as those indicated by the triangular marks, see Figure 8d cf. Figure 4d) after electrochemical polarization tests. According to the phase composition analysis (see Figure 1 and Figure 3) and electrochemical testing results (see Table 2 and Figure 7), nanoscaled flake-like Ca_9_Sr(PO_4_)_6_(OH)_2_ coated Sr-HAC specimens display the optimal coating crystallinity and anti-corrosion performances (much lower corrosion current density, higher corrosion potential and better polarization resistance). Referring to the SEM image of Sr-HAC surface illustrated in Figure 4d, the formation of aggregated nanoscaled flake-like fine crystals on Sr-HAC specimens can provide a firmer protectiveness to prevent direct contact of the SBF solution because of the significantly higher polarization resistance (*R*_p_ = 12.68 kΩ-cm^2^) and lower degradation rate (*P*_i_ = 0.13 mm/year) of the Sr-HAC specimens. As a result, it confirms that the hydrothermally synthesized bioactive HAC and Sr-HAC coatings exhibit better corrosion resistance than alkaline pre-treated Mg(OH)_2_ surface layer. Moreover, this study further demonstrates that the nanostructured bioactive Sr-HAC surface coating behaved as a protection barrier to provide the preferable physiological corrosion resistance for Mg alloys.

### 3.4. Dissolution Behavior Analysis by Immersion Tests

**Figure 9:**
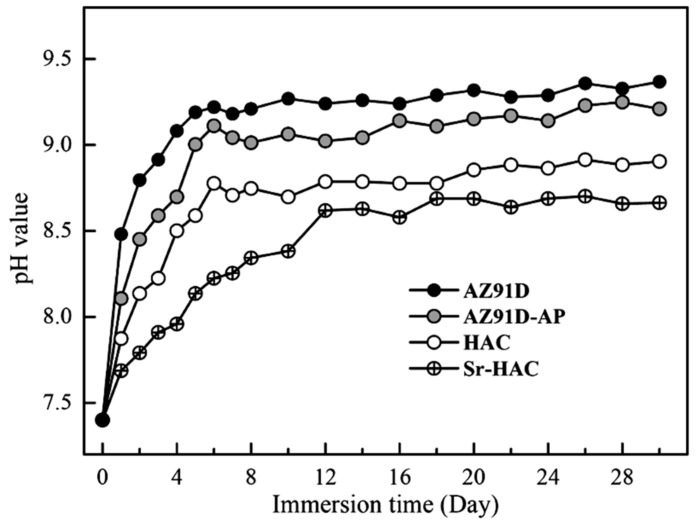
Variation in pH values of the Kokubo’s SBF solution immersed with AZ91D, AZ91D-AP, HAC and Sr-HAC specimens for the immersion duration of 30 days.

**Figure 10:**
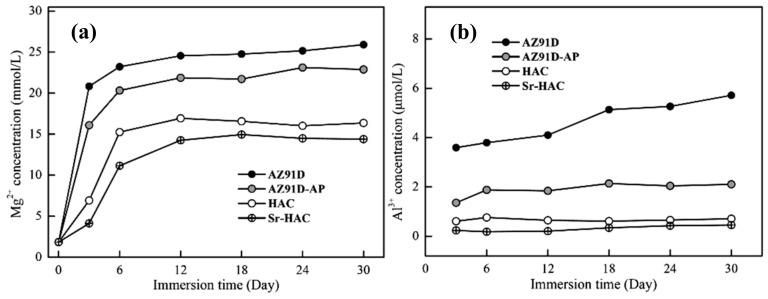
In vitro ion release profiles of (**a**) Mg^2+^ and (**b**) Al^3+^ ion concentration of the SBF immersed with AZ91D, AZ91D-AP, HAC and Sr-HAC specimens for the immersion duration of 30 days.

Figure 9 shows the pH value variation of the AZ91D Mg alloy, AZ91D-AP, HAC and Sr-HAC specimens after immersion tests in the standard Kokubo’s SBF solution for 30 days. The concentration variation of Mg^2+^ and Al^3+^ ions released from various specimens into the SBF is shown in Figure 10. Since Mg is active in nature and Mg(OH)_2_ is slightly soluble in aqueous solutions, the increasing concentration of Mg^2+^ ions results from the continuous corrosion of Mg alloy in the SBF. The corroded product of Mg(OH)_2_ layer with obvious pitting corrosion is insufficient to provide a desirable anti-corrosion performance for the substrate (see Table 2 cf. Figure 8a,b). Therefore, it can be seen that the pH value of immersed AZ91D and AZ91-AP increases rapidly from neutral to alkaline environments in the initial 6 days due to the release of OH¯ ions, and then the pH values for both immersed conditions became stabilized at about 9.3 and 9.1 for longer immersion times (see Figure 9). Meanwhile, the concentration of released Mg^2+^ ions is also significantly increased with the dissolution of Mg substrate in the initial 6 days, and the concentration of Mg^2+^ ions steadily increases to about 25.1 and 22.3 mmol/L for AZ91D and AZ91D-AP specimens, respectively (see Figure 10a). Considering the data of HAC and Sr-HAC specimens as illustrated in Figure 9, however, the increase of pH values is reduced compared to the uncoated AZ91D and alkaline pre-treated AZ91-AP. The pH values for immersed HAC and Sr-HAC specimens are stabilized at about 8.8 and 8.6 after immersion for longer than 6 and 12 days, respectively. In addition, the increasing concentrations of released Mg^2+^ ions for both HAC and Sr-HAC are also obviously lower than that of AZ91D and AZ91D-AP specimens as represented in Figure 10a. The stable concentration of released Mg^2+^ ions in the SBF is about 16.8 and 14.5 mmol/L for the HAC and Sr-HAC specimens, respectively. Figure 10b represents the concentration of Al^3+^ ions released into the SBF solution from various specimens. The concentration of Al^3+^ ions in the SBF released from uncoated AZ91D Mg ally gradually increases from about 3.6 to 5.5 µmol/L with the immersion time. It is noted that the release of Al^3+^ ions is significantly impeded, and the concentration is stabilized at around 0.4 and 0.7 µmol/L during the immersion period for the hydrothermal HAC and Sr-HAC coated specimens, respectively. Referring to the potentiodynamic polarization testing results represented in Figure 7 and Table 2, it is recognized that the dissolution of AZ91D Mg alloy can be effectively reduced with the deposition of hydrothermal coatings. The Sr-HAC coating further enhances better corrosion resistance and promotes long-term stability for the Mg substrate in physiological environments.

**Figure 11:**
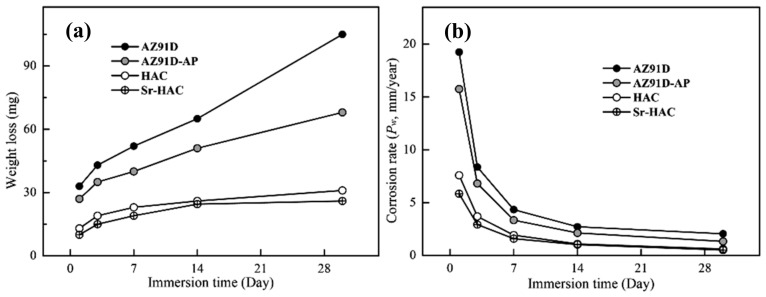
(**a**) Weight loss and (**b**) corrosion rate of the AZ91D, AZ91D-AP, HAC and Sr-HAC specimens evaluated after in vitro immersion tests in the SBF at 37 °C for 1, 3, 7, 14 and 30 days.

Figure 11 represents the weight loss results and corresponding corrosion rates (*P_W_*, mm/year) evaluated after long-term immersion tests in the SBF solution for 1, 3, 7, 14 and 30 days at 37 °C. As shown in Figure 11a, it is observed that the weight losses increase with increasing immersion time for all the specimens. However, the weight losses are obviously reduced and became stabilized for both hydrothermal HAC and Sr-HAC specimens after 14 days of immersion. In addition, the corrosion rates significantly decrease with the prolongation of immersion duration, especially within the initial 3 days as illustrated in Figure 11b. With the extension of the immersion time, the corrosion rates of the specimens are reduced, and the values tend to stable after 14 days of immersion. Compared with the corrosion rate of the naked AZ91D substrate, the reduction percentages of corrosion rates for the alkaline pre-treated AZ91D-AP, hydrothermal HAC and Sr-HAC specimens are about 18.2% to 21.5%, 60.6% to 70.5% and 70.1% to 75.2% during the immersion durations, respectively. The hydrothermal HAC and Sr-HAC specimens shows similar long-term dissolution behaviors; nevertheless, the Sr-HAC still represents slightly lower corrosion rates than the HAC during the whole immersion period. As a result, we can see the average weight loss and corrosion rate of hydrothermal HAC and Sr-HAC specimens are significantly lower than that of AZ91D and alkaline pre-treated AZ91D-AP.

Referring to static contact angle measurements and electrochemical polarization tests (see Figure 6 and Table 2 cf. Figure 11), the super-hydrophilic capillary action arisen from the polar surface chemistry of Mg(OH)_2_ causes a continuous penetration of corrosive SBF solution to the Mg substrate and the release of Mg^2+^ ions (see Figure 10a). Although the corrosion resistance appears to be improved, relatively higher weight loss and corrosion rate are unfavorable results for the alkaline pre-treated Mg(OH)_2_ layer to supply desirable anti-corrosion properties to the AZ91D Mg alloy. According to the results of long-term immersion tests, it is demonstrated that the hydrothermal HAC and Sr-HAC coatings represent much better dissolution resistance to AZ91D Mg substrate than the alkaline pre-treated Mg(OH)_2_.

### 3.5. Cell Responses

**Figure 12:**
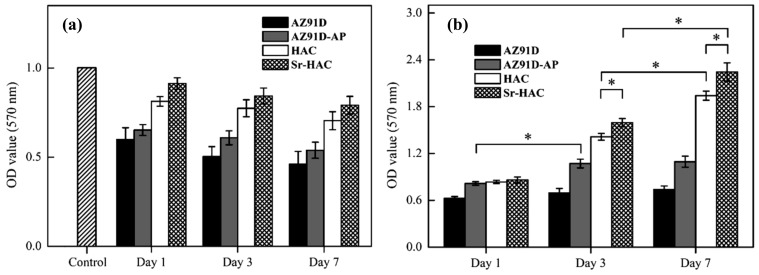
MTT optical density measurements of osteoblastic cell viability for MG63 cells cultured (**a**) in the 1, 3 and 7 days Mg^2+^ conditioned medium for 24 h and (**b**) on the AZ91D, AZ91D-AP, HAC and Sr-HAC specimens for 1, 3 and 7 days. The data are given as the mean ± SD (*n* = 5). The significant difference of groups is labeled according to the one-way ANOVA tests (*: *p* < 0.05).

In order to investigate if cells can survive in a micro-environment with high concentration of Mg^2+^ ions released from AZ91D, AZ91D-AP, HAC and Sr-HAC specimens, Figure 12a shows the cell viability of MG63 cells cultured in various (collected for 1, 3 and 7 days) Mg^2+^ conditioned culture media for 24 h. As shown in Figure 12a, the cell viability is generally decreased within Mg^2+^ conditioned culture media compared to the control. Considering for the AZ91D and AZ91D-AP specimens, it can be recognized that high concentration of released Mg^2+^ ions (about 16–23 mmol/L, see Figure 10a) from the dissolution of Mg substrate results in low cell viability, and the cell viability is gradually reduced with increasing the concentration of released Mg^2+^ ions. Since the concentration of released Mg^2+^ ions is slightly reduced by the alkaline pre-treated Mg(OH)_2_ layer, the cell viability in Mg^2+^ conditioned medium of AZ91D-AP is slightly higher than that of naked AZ91D alloy. Compared to the AZ91D and AZ91D-AP specimens, the concentrations of released Mg^2+^ ions for the hydrothermal HAC and Sr-HAC specimens are obviously reduced to about 6.9–15.3 mmol/L and 4.1–11.2 mmol/L (see Figure 10a) in the initial 6 days, respectively. In addition, the cell viability in Mg^2+^ conditioned medium of hydrothermal Sr-HAC is slightly higher than that of HAC. Referring to the immersion tests, the hydrothermal HAC and Sr-HAC coatings represent better dissolution resistance than the AZ91D and alkaline pre-treated AZ91D-AP specimens. Since the increasing concentrations of released Mg^2+^ ions for both HAC and Sr-HAC are apparently lower than that of AZ91D and AZ91D-AP specimens, the cell viability in Mg^2+^ conditioned media of HAC and Sr-HAC is significantly improved and higher than that of AZ91D and AZ91D-AP specimens (see Figure 12a cf. Figure 10a). As a result of the cell viability within various Mg^2+^ conditioned media illustrated in Figure 12a, it can be recognized that the cell viability of MG63 cells is improved with reducing the concentration of released Mg^2+^ ions in a physiological environment.

The cell viability of MG63 cells on the AZ91D, AZ91D-AP, HAC and Sr-HAC specimens are estimated through the absorbance value from MTT assay after culturing for 1, 3, and 7 days, respectively, as shown in Figure 12b. During the incubation durations, we can see the cell number on the uncoated AZ91D substrate is generally lower than that on the surface-treated specimens. Therefore, it can be recognized that surface treatments with protective coatings can improve the cell viability for biodegradable Mg alloys. After 1 day of culture, the cell numbers in the media of the AZ91D-AP, HAC and Sr-HAC specimens represent no statistically significant differences (*p* > 0.05). After 3 days of culture, the cell numbers on the AZ91D-AP, HAC and Sr-HAC specimens are significantly increased (*p* < 0.05). It is noted that the cell viability on hydrothermal HAC and Sr-HAC specimens are significantly higher than that on the alkaline-pre-treated AZ91D-AP specimens. In addition, the cells cultured on Sr-HAC specimens show slightly higher viability with the difference (*p* < 0.05) than that of cells on HAC specimens. After 7 days of culture, the number of cells cultured on the hydrothermal HAC and Sr-HAC specimens is further increased, and the Sr-HAC specimens still show significantly higher cell viability than the HAC specimens (*p* < 0.05). However, there is no significant difference in the cell viability for the AZ91D-AP between 3 and 7 days of culture.

Referring to the above-mentioned electrochemical corrosion and long-term immersion tests results (see Figure 8, Figure 9 and Figure 10), the alkaline pre-treated Mg(OH)_2_ layer with an obvious pitting corroded surface (as illustrated in Figure 8b) represents limited anti-corrosion performances and long-term dissolution properties. As a result, the Mg(OH)_2_ layer is insufficient to impede the continuous penetration of Mg^2+^ ions released from the degraded AZ91D Mg substrate accompanied with the increase of pH values and the excessive Mg^2+^ ions of surrounding physiological environments. The above-mentioned results illustrated in Figure 12a and relative reports have indicated that the culture medium with high concentration of Mg^2+^ ions is detrimental to the viability, proliferation and growth of cells [21,51]. Therefore, the alkaline medium with abundant released Mg^2+^ ions through the obvious pitting corroded Mg(OH)_2_ structure significantly leads to low viability of MG63 cells on the Mg(OH)_2_ layer (see Figure 9 and Figure 10a cf. Figure 12b).

Considering the immersion testing results of hydrothermal HAC and Sr-HAC specimens, the concentration of released Mg^2+^ ions is effectively reduced and the long-term dissolution behaviors stabilized after 3 and 7 days of immersion (see Figure 10a cf. Figure 11). A recent study has reported that the extracts of surface-coated Mg alloy have no obvious cytotoxicity during the culturing durations [52]. Meanwhile, cells can better tolerate a slightly increase of Mg^2+^ ion concentration in the medium [52]. Since hydroxyapatite is a well-known bioactive ceramic, significantly higher viability of the cells cultured on both of HAC and Sr-HAC can be deduced from their preferable bioactivity and relatively lower released Mg^2+^ ion concentration compared with the AZ91D-AP specimens. Hydrothermally synthesized HAC and Sr-HAC coatings effectively enhance the biocompatibility of biodegradable AZ91D alloy. Considering for the Sr-HAC specimens, it is reported that Sr element plays an important role as a chemical activator to trigger cell responses [41], and the Sr-containing coating allows more continuous direct bone apposition in vivo [53]. Moreover, several reports have also demonstrated that the coating surface with micron/nano-topography can affect the biological responses of osteoblasts and fibroblasts for orthopedic applications [54,55]. The surface coating possesses a topography with nanoneedles significantly enhancing osteoblast cell viability and osteogenic differentiation capacity [48,55]. Referring to the differences in surface chemistry (as illustrated in Figure 3) and surface topography between Sr-HAC and HAC specimens (see Figure 4d cf. Figure 4c), the further enhanced osteoblastic cell viability on Sr-HAC specimens can be ascribed to the substitution of Sr^2+^ ions and the formation of a specific surface topography with nanoscaled crystallites for the Sr-containing HA coating.

## 4. Conclusions

Surface treatments of AZ91D Mg alloy including alkaline pre-treated Mg(OH)_2_, hydrothermal synthesized HAC and Sr-HAC conversion coatings are analyzed in terms of anti-corrosion performances and cell responses for biomedical applications. The following conclusions are summarized based on the above results and discussion:

(1) Sr^2+^ ions can be simultaneously incorporated into HA crystal structure by the hydrothermal synthesis. Moreover, the Sr-substituted HA conversion coating composed of a specific surface topography with the nanoscaled flake-like crystallites can be uniformly constructed on the surface of AZ91D Mg alloy.
(2) The hydrophilicity of AZ91D Mg alloy is obviously improved with the deposition of alkaline pre-treated Mg(OH)_2_, hydrothermal synthesized HAC and Sr-HAC conversion coatings.
(3) The formation of brucite Mg(OH)_2_ prepared by the alkaline pre-treatment is insufficient to give a desirable corrosion resistance and long-term stability in terms of rapid increasing pH values and released Mg^2+^ ion concentration to the Mg alloy immersed in the SBF solution.
(4) Hydrothermally synthesized HAC and Sr-HAC coatings can provide more substantial protection to enhance the corrosion resistance of AZ91D Mg alloy than alkaline pre-treated Mg(OH)_2_. Moreover, the Sr-HAC coating exhibits preferable anti-corrosion performances and lower dissolution rate than the HAC coating in the physiological SBF solution.
(5) The cell viability of MG63 cells can be effectively improved by reducing the concentration of released Mg^2+^ ions through the deposition of hydrothermal HAC and Sr-HAC coatings.
(6) The in vitro cell culture tests reveal that the Sr-substituted HA coating with a nanostructured flake surface topography significantly enhances osteoblastic cell viability and improves the biocompatibility of the Mg alloy.

## Figures and Tables

**Table 1:** The average surface roughness (Ra) and static contact angles of grit-blasted AZ91D, alkaline pre-treated AZ91D-AP, hydrothermal HAC and Sr-HAC specimens.

Surface tests	AZ91D	AZ91D-AP	HAC	Sr-HAC
Ra (μm) ^†^	4.5 ± 0.2	11.5 ± 0.7	6.1 ± 0.5	2.7 ± 0.3
Static contact angle	55°	–	10°	12°

† Values were given as mean ± SD, and each value was the average of five tests (*n* = 5).

**Table 2:** Electrochemical parameters of grit-blasted AZ91D Mg alloy, alkaline pre-treated AZ91D-AP, hydrothermal HAC and Sr-HAC specimens in SBF at 37 °C.

Samples	Corrosion Potential *E*_corr._ (V vs. SCE)	Corrosion Current Density *I*_corr._ (μA/cm^2^)	Polarization Resistance *R*_p_ (kΩ-cm^2^)	Corrosion Rate *P*_i_ (mm/year)
AZ91D	−1.58	96.8	0.75	2.21
AZ91D-AP	−1.42	20.2	3.28	0.46
HAC	−1.05	7.9	9.31	0.18
Sr-HAC	−0.33	5.6	12.86	0.13
